# High *in vitro* survival rate of sheep *in vitro* produced blastocysts vitrified with a new method and device

**DOI:** 10.1186/s40104-019-0390-1

**Published:** 2019-11-01

**Authors:** Sergio Ledda, Jen M. Kelly, Stefano Nieddu, Daniela Bebbere, Federica Ariu, Luisa Bogliolo, Dity Natan, Amir Arav

**Affiliations:** 1 Department of Veterinary Medicine, University of Sassari, Sassari, Italy; 2 South Australian Research and Development Institute, Turretfield Research Centre, 129 Holland Road, Rosedale, SA 5350 Australia; 3 FertilSafe Ltd, 11 Haharash st, 7403118 Ness Ziona, Israel

**Keywords:** Blastocysts, High survival, In-straw, *In vitro* embryo produced, Ovine

## Abstract

**Background:**

To advance the use of embryo vitrification in veterinary practice, we developed a system in which embryo vitrification, warming and dilution can be performed within a straw. Ovine *in vitro* produced embryos (IVEP) were vitrified at either early (EBs: *n* = 74) or fully expanded blastocyst stage (FEBs: *n* = 195), using a new device named “E.Vit”, composed by a 0.25-mL straw with a 50-μm pore polycarbonate grid at one end. Embryos at each stage (EBs and FEBs) were vitrified by either Two-step (TS) or Multi-step (MS; 6 different concentrations of vitrification solutions) protocol. Non-vitrified embryos (*n* = 102) were maintained in *in vitro* culture as a control. Warming consisted of placing the straws directly into 1.5 mL tubes containing a TCM-199 solution with three decreasing concentrations of sucrose. Blastocyst re-expansion, embryo survival and hatching rate were evaluated at 2, 24 and 48 h post warming. The number of apoptotic cells was determined by TUNEL assay.

**Results:**

Blastocyst re-expansion (2 h) after warming was higher (*P* < 0.05) in FEBs group, vitrified with the MS and TS methods (77.90% and 71.25%, respectively) compared with the EBs group (MS: 59.38% and TS: 48.50%, respectively). Survival rates of vitrified FEBs after 24 h IVC were higher (*P* < 0.001) in both methods (MS and TS) than vitrified EBs (MS: 56.25%; TS: 42.42%) and was higher (*P* < 0.05) in the MS method (94.19%) compared with those in TS (83.75%). After 48 h of culture the hatching rate for FEBs vitrified in MS system (91.86%) was similar to control (91.89%), but higher than FEB TS (77.5%) and EBs vitrified in MS (37.5%) and TS (33.33%). Number of apoptotic cells were higher in EBs, irrespective of the system used, compared to FEBs. The number of apoptotic cells in FEBs vitrified with MS was comparable to the control.

**Conclusions:**

A high survival rate of IVP embryos can be achieved by the new “E.Vit” device with hatching rates *in vitro* comparable with control fresh embryos. This method has the potential for use in direct embryo transfer in field conditions.

## Introduction

Over the past decades, the worldwide increase in small ruminant breeding has been supported by the development and improvement of assisted reproductive technologies (ART) [1, 2]. However, while some ART including estrus induction, estrus synchronization and artificial insemination (AI) have reached widespread application, the adoption of ART, such as superovulation and embryo transfer (MOET), *in vitro* embryo production (IVEP) and embryo cryopreservation have, to date, been limited.

New prospects offered by IVEP, repeated ovum pick-up from live adult and juvenile female donors, suggest that IVEP technology can be used as an alternative system to MOET programs, thus moving the use of this technology from exclusive research in the laboratory to the field [3]. Recent improvements of embryo production and cryopreservation technologies have the potential to allow wider propagation of valuable genetics in small ruminant populations and establishment of flocks without risk of disease transmission. In addition, these technologies could make a substantial contribution to the preservation of endangered species or breeds.

Embryo cryopreservation has become an integral part of the commercial embryo transfer industry, but its application in small ruminants is still limited [4, 5]. From the practical viewpoint, embryo cryopreservation, has many advantages, it facilitates i) distribution of superior genetics from dams of high genetic value, which accelerates the rate of genetic improvement and ii) international transportation of valuable genetic stock, which is a financially feasible and safe alternative to live animal transport. To date, documented use and success rates for different cryopreservation techniques and devices in small ruminants are relatively scarce compared with cattle [6–8]. However, it has been observed that sheep embryos are able to survive both “equilibrium cooling” or controlled slow freezing and vitrification procedures [9].

Controlled slow freezing protocols require a biological freezer and need more time to be completed, while the ultra-rapid techniques such as vitrification require no special equipment and are time and cost effective, therefore, making this technology more adapted to routine field use [4]. Moreover, the viability rate for cryopreserving *in vitro* and *in vivo* derived morula and blastocyst stage ovine embryos by vitrification is significantly higher compared with embryos cryopreserved by slow freezing techniques [10–12].

Different factors can affect the efficiency of the cryopreservation system used for embryo freezing. The origin of the embryo, *in vivo* vs. *in vitro*, contributes to these differences with *in vivo* produced embryos having increased viability and survival rates post thawing compared with their *in vitro* counterparts [13, 14]. In addition, several methods and devices have been proposed in order to improve and simplify embryo cryopreservation methodologies of the different farm species [15–17]. In ovine, the 0.25 mL straw [18] and the open pulled straw (OPS) have been successfully used for vitrifying *in vivo* [4, 9] or *in vitro* [5] produced morula and blastocysts.

The successful application of IVEP embryo cryopreservation and transfer in small ruminants is largely dependent on the efficiency of the techniques used.

Vitrification has become a viable and promising alternative to traditional slow freezing approaches since is simple, rapid and fewer equilibration and cryopreservation steps are needed. For the use of vitrification technology in veterinary practice, embryos would have to be warmed and diluted in the vitrification straw so that they could be directly transferred to the uterus of recipients animals. Several methods have been designed to facilitate the direct transfer of vitrified embryos in field conditions with a different level of complexity of the technical procedures [19, 20].

In this vein, we are proposing a new system, “E.Vit” in which embryo vitrification, warming and dilution can be performed within a straw. An in-straw embryo cryopreservation method reduces the need for equipment, technical skills and embryo handling and can facilitate direct embryo transfer to the uterus.

To evaluate the feasibility and efficiency of the new “E.Vit” system, we compared the post-warming survival rate of IVEP embryos (early or expanded blastocyst) vitrified by Two- or Multi-step systems with fresh IVEP embryos (control).

## Materials and methods

### Chemical

All chemicals were purchased from Sigma-Aldrich Chemical Co. unless otherwise specified.

### Oocytes recovery

Ovaries from adult Sardinia sheep (*Ovis Aries*, 3-6 years old) were recovered from local abattoirs and transported within 3 h to the laboratory in Phosphate Buffered Saline (PBS) with penicillin (100 mg/mL) and streptomycin (100 mg/mL) at 27–30 °C.

Upon arrival at the laboratory, ovaries were cleaned of any tissue residues, washed twice in PBS and placed in Holding Medium (HM) at pH 7.22 ± 0.1 at 37 °C consisting of TCM-199 with Hepes (N-2-Hidroxyethylpiperazine-N-2-ethansulfonic acid) 25 mmol/L, 50 IU/mL of antibiotics (streptomycin and penicillin), sodium bicarbonate 0.005 mol/L and 0.1% (*w*/*v*) of polyvinyl alcohol (PVA). The ovaries were divided sagittally with the aid of a sterile microblade and cumulus oocyte complexes (COCs) were released from the follicles by slicing technique.

### *In vitro* maturation (IVM), *in vitro* fertilization (IVF) and *in vitro* embryo culture (IVC)

For *in vitro* maturation (IVM), COCs with several intact cumulus cells layers and homogenous cytoplasm were selected. COCs were matured in 650 μL of *in vitro* maturation medium consisting of TCM-199 containing 10% heat-treated estrous sheep serum (ESS), 0.36 mmol/L pyruvate, 100 mmol/L cysteamine, FSH 1 IU/mL and LH 1 IU/mL (Pluset; Bio98, Milan, Italy) under mineral oil, in 4-well dishes (Nunc Cell Culture, Thermo Fisher Scientific, Waltham, Massachusetts, USA) in a humidified atmosphere of 5% CO_2_, at 38.5 °C.

After 24 h from IVM, COCs were partially stripped of cumulus cells as described by Bogliolo and co-authors [21] and co-incubated with frozen-thawed spermatozoa, selected by the swim-up technique, (1 × 10^6^ spermatozoa/mL) in IVF medium consisting of Synthetic Oviductal Fluid (SOF) [22] supplemented with 2% ESS, 1 g/mL heparin, 1 g/mL hypotaurine for 22 h at 38.5 °C in a humidified atmosphere of 5% CO_2_, 5% O_2_, 90% N_2_ in four well Petri dishes.

The semen was preliminarily evaluated with the aid of the stereomicroscope (mass motility) and of the CASA system (Ivos, Hamilton Thorne, Biosciences).

At the end of IVF (approximately 24 h), presumptive zygotes (*n* = 15-20) were transferred to 650 μL IVC medium (SOF supplemented with BSA (4 mg/mL), essential amino acids (EAA) and non-essential amino acids (NEAA) at oviductal concentration [23] in 4-well culture dishes and incubated at 38.5 °C under 5% O_2_, 5% CO_2_, 90% N_2_ and maximum humidity. At 30 h post fertilization cleavage rate was recorded and on day 6 and 7 (day 0 = day of IVF) embryos were evaluated for development.

### Embryo vitrification

**Fig. 1:**
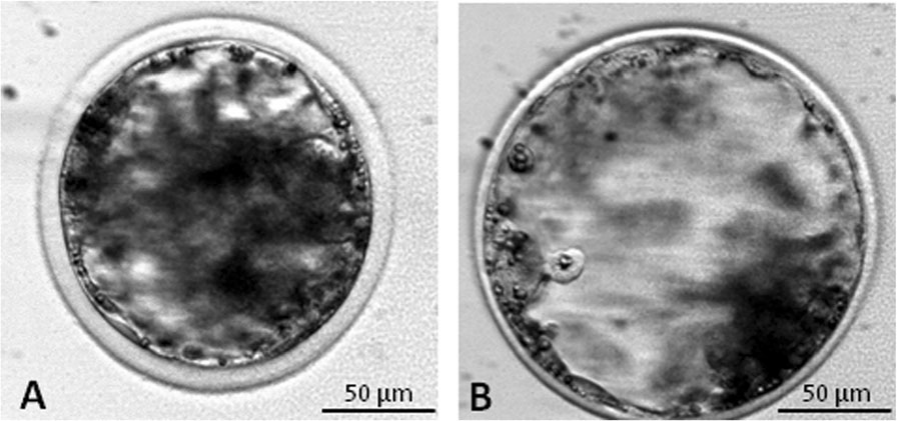
Representative images of *in vitro* produced embryos at different development stages: **a** Early blastocyst (EB); **b** Expanded blastocyst (FEB)

For the experiments of vitrification, blastocysts were classified according to degree of expansion and hatching status as: early blastocyst (EB: blastocyst with a blastocoel less or equal to the half of the embryo volume), fully expanded blastocyst (FEB: a large blastocyst with a blastocoel greater than half of the embryo volume) (Fig. 1).

**Fig. 2:**
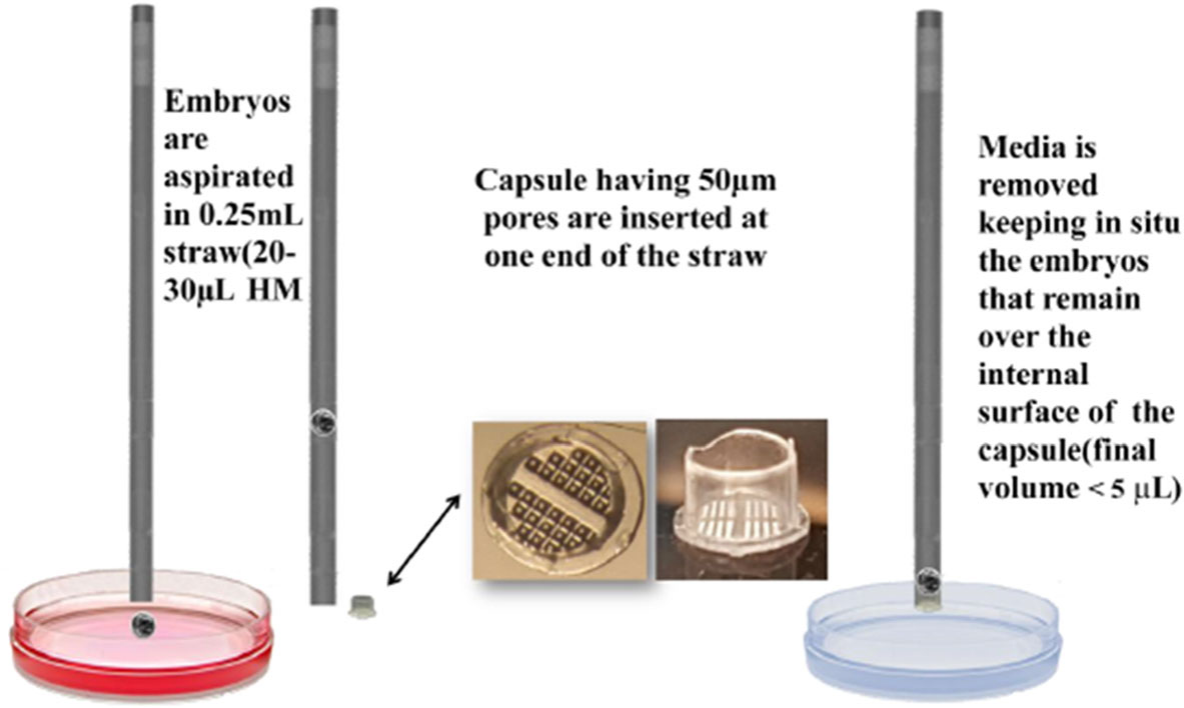
Sample insertion into E.Vit device

The “E.Vit” device (FertileSafe, Ness Ziona, Israel) used for vitrification consisted of a 0.25-mL straw with a polycarbonate grid (capsule) with pore diameters of 50 μm inserted at free end of the straw (Fig. 2). At day 6 or 7 of IVC either EB or FEB were placed in embryo handling medium (EHM see below) before being loaded by aspiration into the straw.

**Fig. 3:**
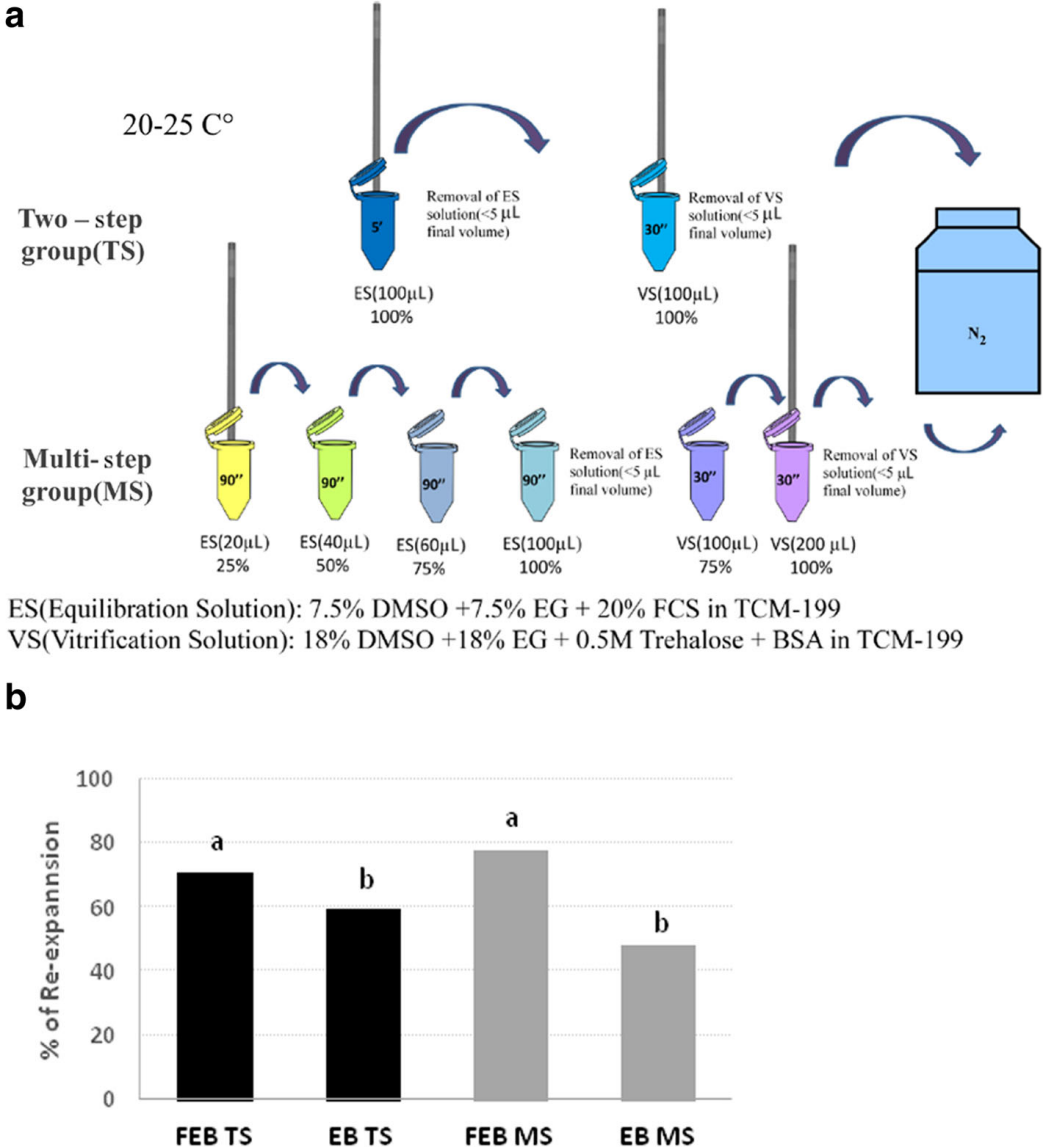
Flow chart of blastocyst vitrification using E.Vit device. **a** Two-step method **b** Multi-step method

Each 0.25 mL empty straw was loaded with up to 2 embryos in a volume range of 20–30 μL, taking care to leave an air bubble at the end of straw. Next, the capsule was inserted by pressure into the straw end (Fig. 2). The insertion of the capsule allowed the removal of excess HM medium; the solution that contains the embryo is reduced to 5 μL. The straw loaded with the embryos and with the inserted capsule is transferred between the following solutions (Fig. 3); the volume of the solution in the straw is increased by capillary force and according to the increasing volume of the different vials (Fig. 3). The straws are evacuated by gentle blotting on sterile gauze in two times: the first after loading the 100% ES and the second after loading with 100% VS solution.

Three replicates of each embryonic stage and vitrification system were performed*.* The experiment was performed at room temperature (20-25 °C) and all media were at 25 °C using either:

- **Two-step system (TS)**, with the exposure of the embryos at only one equilibration and one vitrification solution or;
- **Multi-step system (MS)**, based on the exposure to 4 increasing concentrations of cryoprotectants for the equilibration procedure and two different concentrations for the vitrification solution.

*Embryo handling medium (EHM)* consisted of TCM-199 + HEPES supplemented with 0.6% bovine serum albumine (BSA) and 0.5 mol/L trehalose.

*Equilibrium solution (ES)* consisted of EHM supplemented with 7.5% of ethylene glycol (EG) and 7.5% of dimethyl sulphoxide (DMSO) .

*Vitrification solution (*VS*)* consisted of EHM supplemented with 18% of EG and 18% DMSO.

### Two-step system (TS) (Fig. 3a)

The TS involves the use of a single equilibrium solution (ES: 100%) and a single vitrification solution (VS: 100%). The embryos were loaded into the E.Vit device (as described above) and then the E.Vit device lowered into ES (1 mL of ES in 1.5 mL micro-centrifuge tube) for 5 min. After which the ES was gently removed from the E.Vit device before being lowered into VS (1 mL of VS in 1.5 mL micro-centrifuge tube) for 30 s and then plunged into liquid nitrogen. Between the passage from the VS and the immersion in liquid nitrogen, excess VS was quickly removed by gentle blotting on sterile gauze, from the E.Vit device.

### Multi-step system (MS) (Fig. 3b)

After loading embryos into the E.Vit device (described above), excess handling medium was removed from the E.Vit device before being exposed, in a step-wise manner, to increasing concentrations of ES cryoprotectants (1 mL of each of the following ES concentrations diluted with EHM) set up in 1.5 mL micro-centrifuge tubes; ES 1: 25%, ES2: 50%, ES3: 75%, ES4: 100%). The E.Vit device with the embryos was exposed to each ES concentration (with increasing volume) for 90 s for a total of 6 min. Between the equilibrium phase and the exposure to the VS1 medium, excess ES4 was removed from the E.Vit device by gentle blotting on sterile gauze. The E.Vit device containing the embryos was subsequently plunged into 1 mL of 75% of final VS concentration (VS1) for 30 s (1.5 mL micro-centrifuge tube), followed immediately by plunging into 100% VS concentration (VS2) for 30 s (1 min total exposure for the two VS concentrations). Excess V2 medium was removed from the E.Vit device by gently blotting on sterile gauze before being immersed into liquid nitrogen.

### Warming of the embryos

Embryos were left in LN_2_ storage for a minimum 7 days. Warming for both the TS and MS systems was carried out as follows. The E.Vit device, with the embryos inside, immediately after removing from the liquid nitrogen, was plunged stepwise into decreasing concentrations of sucrose (1 mol/L, 0.5 mol/L and 0.25 mol/L in TCM-199 + 20% FCS at 38.5 °C). The E.Vit device was left immersed in each solution for 5 min.

#### Embryo recovery and post warming *in vitro* culture

After the warming procedure, the capsule was removed by cutting the end of straw and embryos released in HM. The number of embryos recovered after the removing the capsule from each straw was recorded. The embryos were washed 2-3 times with HM and incubated in IVC at 38.5 °C at 5% CO_2_ at maximum humidity for the subsequent evaluations after 2, 24 and 48 h of culture.

For the evaluation we have used the following morphometric criteria [24]:

- Time of the start of re-expansion (the first appearance of the blastocoele cavity or increase in size).
- Time of completion of re-expansion (the blastocyst occupies the whole perivitelline space).
- Time of hatching (the trophectoderm blebbs out of the zona pellucida).

In particular, the following embryo development parameters were recorded:

- after 2 h: number of embryos that showed a start of re-expansion of the blastocoel cavity;
- after 24 h: number of embryos with completion of expansion of blastocoel cavity;
- after 48 h: number of hatched blastocyst.

### Analysis of apoptosis through the use of the TUNEL technique and confocal microscope

Apoptotic cell death in the embryos was evaluated by TUNEL using an In situ Cell Death Detection kit reaction mixture (Fluorescein; Roche Diagnostics Corp., Indianapolis, IN, USA). This method is used to detect cells that contain single and double strand breaks (nick) more or less extensive along the nuclear DNA molecule.

The TUNEL kit consists of an enzyme, the TdT (Terminal deoxynucleotidyltransferase), which catalyzes the polymerase reaction of nucleotides (always added to the mixture) labeled with fluorescein [25], at the free end 3´OH of fragmented DNA molecules both at the single and double stranded levels. The labeled nucleotides “fill” the single or double strand breaks on the DNA and emit a typical fluorescence green light; the more intense the more the DNA breaks are extended. To determine apoptosis of embryos a subset of embryos from Control fresh IVP embryos (EB *n* = 13; FEB *n* = 15), and from vitrified/warmed TS (EB *n* = 14; FEB *n* = 12) and MS (EB *n* = 12; FEB *n* = 20) after 24 h of IVC were fixed in 4% paraformaldehyde in PBS at 37 °C for 1 h. Following fixation, they were washed 3 times per 15 min in PBS + 0.1% PVA, to remove the residual fixative. The embryos were then permeabilized with 0.1% Triton X 100 in 0.1% sodium citrate for 5 min at 4 °C. The permeabilization aims to make permeable the zona pellucida at the entrance of the reagents that will be added later. The samples were incubated in TUNEL (Enzyme Solution + Label Solution) for 1 h at 38.5 °C in the dark. As a negative control, embryos (EB *n* = 2; FEB *n* = 3) from the control group were incubated in the presence of Label Solution and subsequently processed, as described below for the other groups of vitrified embryos.

Following incubation, the embryos were washed 3 times for 15 min in PBS+ 0.1% PVA, stained with a Glycerol-Hoechst 33342 solution (10 μg/mL) for the evaluation of the nuclear chromatin of the blastomeres. A drop of dye solution was placed on a glass slide and the embryos were transferred (3-5 embryos) to the drop. A coverslip was placed on the drop to slightly compress the embryos in order to facilitate the visualization of the nuclei. The slide was kept for 1 h in the dark at 4 °C and then read with the confocal microscope. The images were acquired with a laser scanning confocal microscope (Leica TCS SP5), equipped with 543 nm HeNe, 488 nm Argon and 405 nm 405-diode laser using an immersion objective (in oil) 40 × (NA = 1.25). The parameters related to fluorescence intensity (laser energy, gain, offset and pinhole size) were maintained with constant values during all the acquisitions of images.

TUNEL’s green fluorescence was determined using excitation wavelengths of 488 and 543 nm and emission spectra of 515-565 nm (green).

The number of TUNEL positive nuclei in embryos was determined and the apoptotic index (No. of apoptotic cells/ total No. of cells × 100) was calculated [26].

### Statistical analysis

Data were analyzed by StataIC 11.2 software (Stata Corp LP, USA). Recovery rate, blastocoel re-expansions after 24 h post warming and hatching (survival) after 48 h post warming were compared between embryonic stage EB and FEB and between the two methods of vitrification procedures (TS and MS) using Chi-square χ^2^ test with *post hoc* Bonferroni test. Values of *P* < 0.05 were considered statistically different.

## Results

### Embryo recovery

The embryo recovery rates from the “E.Vit” system are reported in Table 1 comparing the different embryonic stages (EB vs. FEB) and the different methods of cryoprotectant exposure (TS vs. MS). No statistically significant differences were observed between the embryonic stages and methods of cryoprotectant exposure.

**Table 1:** Recovery rates of early blastocyst (EB) and fully expanded blastocyst (FEB) vitrified using E.Vit device with Multi-step or Two-step methods

	Embryonic stage	No. of embryos	No. of recovered embryos	Recovery rate, %
Two-step (TS) method	EB	37	33/37	89.1%
	FEB	90	80/90	88.89%
Multi-step (MS) method	EB	37	32/37	86,49%
	FEB	95	86/95	90.5%

### Blastocoel re-expansion after 2 h post warming *in vitro* culture

**Fig. 4:**
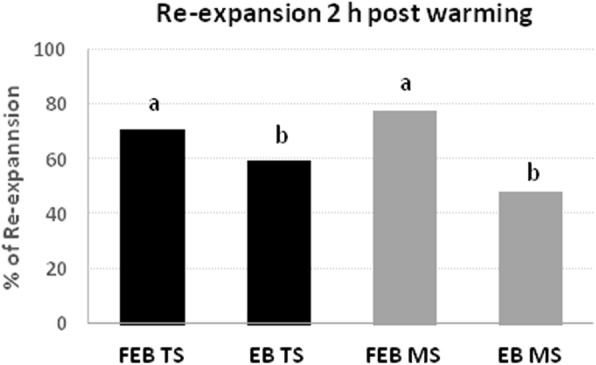
Rate of post warming blastocoel re-expansion of EB and FEB blastocysts vitrified in “E.Vit” device with Two-step (TS) and Multi-step (MS) method. Different letters above columns indicate significant differences (*P* < 0.05; Chi-square test). Survival rates of vitrified early (EB), expanded blastocysts (FEB) by Two-step (TS) and Multi-step (MS) methods and control (CTR) after culture *in vitro* for 24 h. Different letters above columns indicate significant differences (*P* < 0.05; Chi-square test)

After 2 h post warming, overall rates of embryos that showed a regular blastocoel re-expansion were not affected by TS or MS vitrification methods. However, a significant difference (*P* < 0.05) was observed between EB [TS: *N* = 16/33 (48.5%) and MS: *N* = 19/32 (59.38%)] and FEB [TS: *N* = 57/80 (71.25%) and MS: *N* = 67/86 (77.91%)] within both methods (Fig. 4).

### Survival of embryos after 24 h of post warming *in vitro* culture

**Fig. 5:**
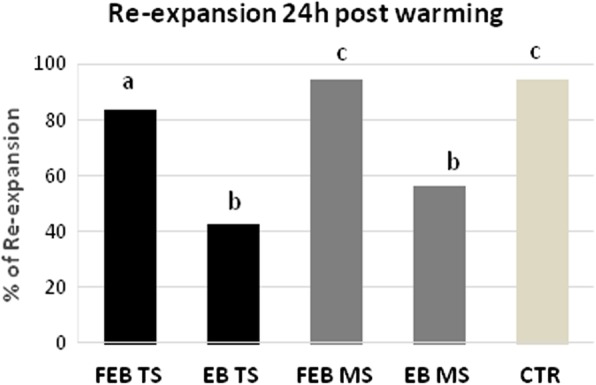
Survival rates of vitrified early (EB), expanded blastocysts (FEB) by Two-step (TS) and Multi-step (MS) methods and control (CTR) after culture *in vitro* for 24 h. Different letters above columns indicate significant differences (*P* < 0.05; Chi-square test)

Survival rates were significantly (*P* < 0***.***001) higher after vitrification of fully expanded blastocysts (FEB) compared with the early blastocyst (EB) in the TS method [FEB: *N* = 67/80 (83.75%) vs EB: *N* = 14/33 (42.42%), respectively] and in the MS method [FEB: *N* = 81/86 (94.19%) vs EB: *N* = 18/32 (56.25%), respectively). Rate of survival was also significantly different between the FEB vitrified in TS method compared with the MS method (*P* = 0.031). The non-cryopreserved control (CTR: *N* = 70/74; 94.59%) differed from all vitrified-warmed groups except for FEB (*N* = 81/86; 94.19%) vitrified with the MS method (Fig. 5).

### Hatching rate after 48 h post warming *in vitro* culture

**Fig. 6:**
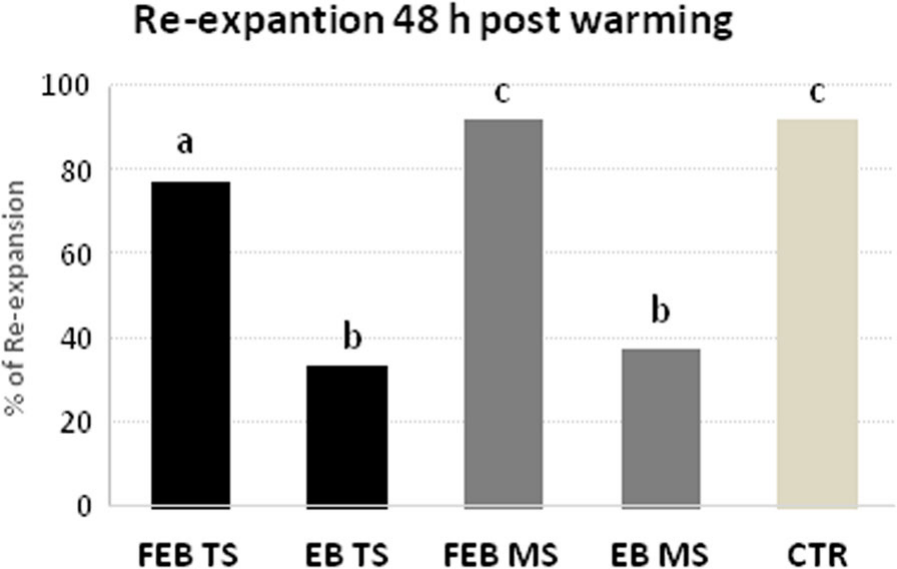
Hatching rates of vitrified early (EB) and expanded blastocysts (FEB) by Two-step (TS) and Multi-step (MS) methods and control (CTR) after *in vitro* culture for 48 h. Different letters among columns indicate significant differences (*P* ≤ 0.01; Chi-square test)

After 48 h of culture the hatching rates were higher in the vitrified fully expanded embryos (FEB), irrespective of the system used, compared with early blastocysts [FEB TS: *N* = 62/80 (77.5%) vs EB TS: *N* = 11/33 (33.30%) and FEB MS: *N* = 79/86 (91.86%) vs EB MS *N* = 12/32 (37.50%); *P* < 0.001 in both cases]. The hatching rate of vitrified FEB with the MS method was higher (*P* = 0.01) compared with FEB for the TS method and similar to controls (*N* = 68/74; 91.89%; Fig. 6).

### Apoptotic cell detection in vitrified and control blastocysts

**Fig. 7:**
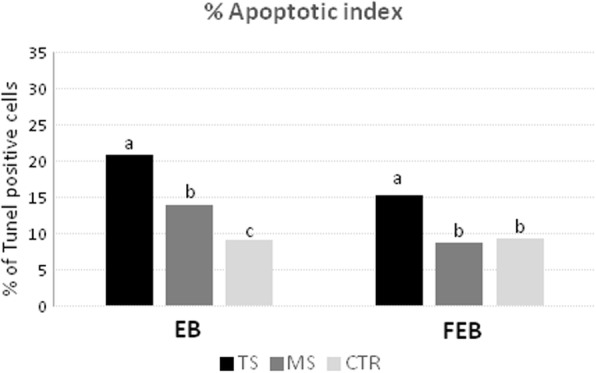
Percentage of apoptotic index (TUNEL) in early (EB) and expanded blastocysts (FEB) for control (CTR) and Two-step (TS) and Multi-step (MS) vitrification methods. Different letters represent statistical difference (*P* < 0.001)

The results of DNA fragmentation evaluated by TUNEL technique using the two vitrification systems are shown in Fig. 7.

The apoptosis index in vitrified EBs was higher in TS (26.11%) compared with the MS (16.48%) and control group (9.95%; *P* < 0.001; Fig. 7).

FEBs cryopreserved with the MS method showed apoptosis index (8.95%) significantly lower (*P* < 0.001) than those vitrified with TS method (26.67%) and similar to the Control group (10.28%; Fig. 7).

**Fig. 8:**
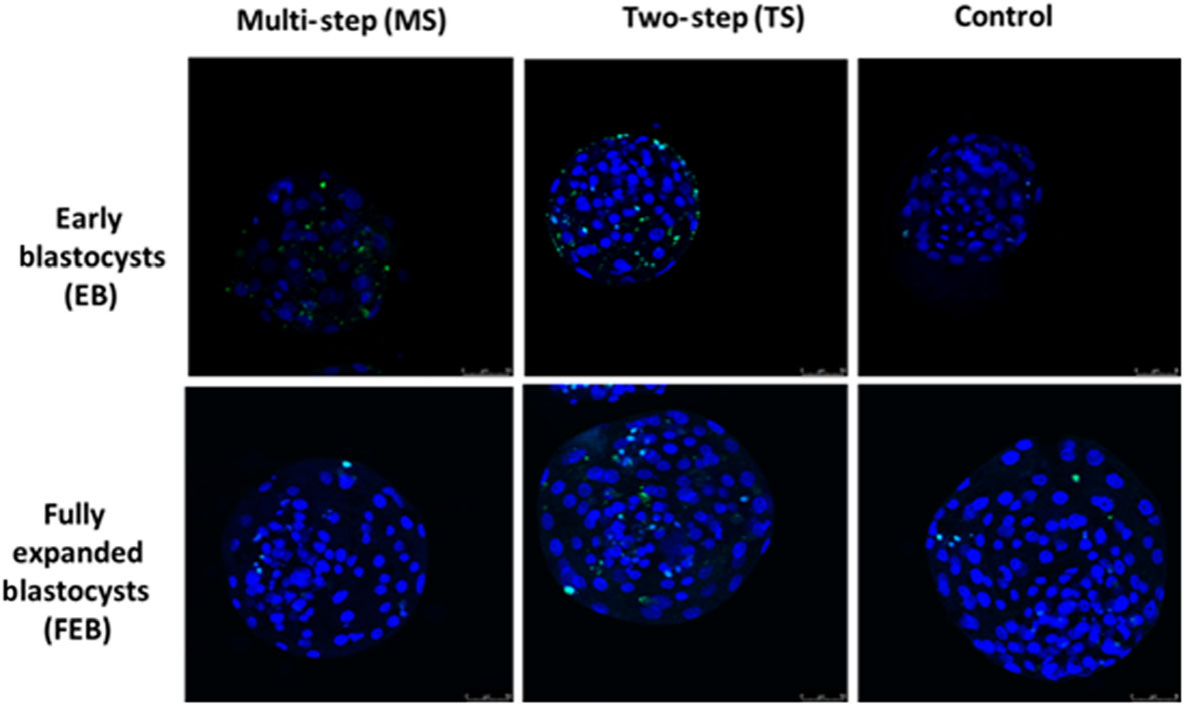
Apoptosis was assessed by TUNEL assay in EB and FEB blastocysts vitrified with TS and MS methods and control. Images of representative apoptotic cells were detected by TUNEL (green) and DNA was stained by DAPI (blue) to visualize all cells

Confocal microscopy analysis allowed for identification of the positive fluorescent signal in the EB, FEB and CTR groups (Fig. 8).

## Discussion

Although cryopreservation of preimplantation embryos has made great progress in recent years, the achievements obtained for ovine embryo freezing, especially for *in vitro* produced embryos are still relatively low. The development of simplified and reproducible vitrification cryopreservation systems that allow gametes, embryos, and reproductive tissues to be successfully cryopreserved are important considerations if this technology is to be widely adopted. Moreover, to achieve a large application of vitrification technology in the field, the need to manipulate embryos should be reduced to a minimum and be able to be performed by operators without a need for specialized skills and equipment.

Here, we report a new vitrification protocol and device (E.Vit) that allowed *in vitro* produced ovine embryos to be efficiently vitrified and thawed in-straw. Our results showed that a step-wise exposure to cryoprotectants during vitrification positively affected embryo survival rates and that embryonic stage at vitrification correlated to embryo survival after vitrification/warming. In addition, our results demonstrated that after vitrification of fully expanded blastocysts (FEB) in multi-step (MS) cryoprotectant exposure, no differences in the survival and hatching rates were observed between vitrified and fresh IVEP embryos after culture *in vitro*. Several factors can affect the efficiency of cryopreservation of ovine *in vitro* produced embryos; among them, embryo survival after cryopreservation has been reported as mainly related to the source of embryos and to the methods used for the embryo freezing [18, 27].

The reduction of survival after cryopreservation of IVP embryos is also due to osmotic and toxic effects exerted by the cryoprotectant exposure. This is more evident in the vitrification procedures that require higher cryoprotectant concentrations. A correct balance between the formulation of cryoprotectant mixture, their concentration, time of exposure and temperature seems also crucial to reduce cryodamage [28]. The right interconnection of these factors could have more importance when chilling sensitive embryos, as the IVP ones, are subjected to cryopreservation. To reduce toxicity and improve osmotic response to cryoprotectants, usually embryos are pretreated / equilibrated in a solution containing a lower concentration of permeating cryoprotectants before being suspended in a vitrification solution. The pretreatment is effective to promote permeation of the cryoprotectant and at the same time reduce toxicity. The concentration of the permeating cryoprotectants used in the pretreatment ranges from 2% to 20%. Our results are in this lane and we have observed that a gradual exposure to increasing concentrations of cryoprotectants before suspending in the final vitrification solution increased the survival rates of vitrified expanded blastocysts, after 24 h of post warming culture. Exposure of the same stage embryos to only one equilibration solution (ES 100%) yielded a significantly lower number of blastocysts able to complete blastocoel re-expansion. The reduction of potential adverse toxic effects depends not only on cryoprotectant solution concentrations and the type of cell, but is also related to temperature. In our experiments, besides using a combination of ethylene glycol (EG) and dimethyl sulfoxide (DMSO), which have high penetration rate and low toxicity, we performed all procedures at room temperature to further reduce the toxicity of cryoprotectants. A similar approach has been reported in previous studies for the vitrification of ovine [5] and bovine embryos [29].

Further key elements for embryos survival by reducing chilling injury are high cooling and warming rates, which are achieved by using a small-volume and a small-size carrier. Various methods were developed to achieve these features, including straws [4], electron microscope grids [30], fine capillaries [31, 32], cryotops [33], cryoloops [34] or the tips of micropipettes [35]. The E.Vit system offers similar advantages in term of cooling rate. It has been previously reported [28] that cooling and warming rates are very fast and similar to other largely used open systems [33–35]. The efficacy of embryo vitrification can be estimated by recording embryo morphokinetics after post warming culture. Previous studies have indicated that, following embryo vitrification/warming, blastocoel re-expansion and hatching is predictive of subsequent development, including ability to implant, establish a pregnancy and result in a live offspring [24, 36]. In addition, timing of blastocoelic cavity re-expansion after vitrification/warming and *in vitro* culture is considered a reliable index of *in vitro* produced embryo quality and developmental potential [37]. Our results showed that embryonic stage can affect the start of re-expansion (2 h post warming), completion of re-expansion (24 h post warming) and hatching rate (48 h post warming). Fully expanded blastocysts consistently yielded better rates compared with early blastocysts, irrespective of the system used, and after 24 h of culture, we observed a higher survival rate of fully expanded blastocyst compared with those vitrified with the two-step (TS) protocol. The fully expanded blastocysts exposed to the MS cryoprotectant protocol and vitrified using E.Vit, gave a hatching rate comparable with fresh control embryos. It has been previously reported that early stages embryos are more sensitive to cryopreservation procedures and that their survival is significantly lower than embryos vitrified at later stages. The proportion of embryos that can survive after cryopreservation is in fact significantly lower in 2-8 cell stage embryos compared with morula and blastocyst stage [10, 12]. Here, we reported that significant differences can be also observed comparing vitrification of early blastocyst stage respect with expanded blastocyst stage. Similarly, a higher survival rate has been observed in ovine *in vivo* [18] and *in vitro* [12] derived embryos vitrified in later stages compared with earlier stage embryos.

The higher cryotolerance of expanded blastocysts could be due to several factors: the cellular membranes of embryos becomes more resistant to osmotic, toxic and chilling stress after the formation of the blastocoelic cavity; the diversification of cell types and in particular the increase of Na^+^/K^+^ ATPase activity, which occurs during blastocoelic formation in trophoblastic cells, may determine more active transport mechanisms of cryoprotectants [38]. Other aspects that can influence the hatching rate may be explained by differences in blastomere size. The cells of compacted morulae and early blastocysts are slightly larger than the cells of expanded blastocyst, and this may render them more sensitive to the osmotic stress induced by the removal of the permeated cryoprotectant [39]. Our results indicated that using the E.Vit device and the MS method resulted in embryo survival and hatching rates comparable with fresh embryos cultured in the same conditions. Gradual exposure using the MS procedure may reduce osmotic stress by allowing water permeability in the same rate of CPs permeability. According results on embryo quality were obtained by the quantification of apoptotic cells. Early vitrified embryos, irrespective of the method used, contained a higher number of apoptotic cells compared with expanded blastocysts. Expanded blastocysts vitrified with the MS method had comparable numbers of apoptotic cells as with the control fresh embryos.

Finally, the extensive use of embryo transfer technology in sheep needs the availability of simple systems that will not require expensive instruments and specialized skills. For this reason previous studies have been carried out to propose in-straw systems that permit the direct transfer of cryopreserved embryos into recipient animals. These systems, in most of the cases, involved more than one device and were developed mainly to perform in-straw warming and dilution [19, 20, 31, 32, 40, 41].

The E.Vit is a simple, efficient, standardized and reproducible vitrification system. In addition to the obvious advantage that all vitrification procedures can be performed in the E.Vit device, is the added benefit that the E.Vit is designed to be used in automated systems [28]. Previous experiments employing mouse embryos, bovine oocytes and ovine testicular tissue indicated the feasibility of the device and the high survival rate after warming [28, 42, 43]. Further advantages include the possibility to employ CE certified 0.25 mL straws, to vitrify many straws simultaneously, to reduce osmotic and mechanical damages and possible contamination during embryo manipulation and to achieve rapid cooling and warming rates (> 20,000 °C/min).

## Conclusions

In conclusion, vitrification of *in vitro* produced ovine embryos by the E.Vit device and multistep cryoprotectant exposure method yielded post warming, high survival rates and embryo quality that could be predictive of subsequent development. The new vitrification protocol and cryodevice can permit that all the procedures of equilibration, cryopreservation as well warming and dilution can be performed in the vitrification straw. This method has the potential for use in direct embryo transfer in field conditions.

Future experiments are under evaluation to establish the full survival potential after embryo transfer in recipient ewes.

## Data Availability

All data generated or analysed during this study are included in this published article.

## Abbreviations

ART: Assisted reproductive technologies
CASA: Computer-assisted sperm analysis
CE: European Community
CLA: *Trans*-10*cis*-12-conjugatedlinoleumacid isomer
COC: Cumulus oocyte complexes
CTR: Non-cryopreserved control
DMSO: Dimethyl sulphoxide
EAA: Essential amino acids
EB: Early blastocysts
EG: Ethylene glycol
EHM: Embryo handling medium
ES: Equilibrium solution
FCS: Fetal calf serum
FEB: Fully expanded blastocysts
FSH: Follicle stimulating hormone
HM: Holding Medium
IVEP: *In vitro* embryo produced
IVEP: *In vitro* produced embryos
IVF: *In vitro* fertilization
IVM: *In vitro* culture
IVM: *In vitro* maturation
MOET: Multiple ovulation and embryo transfer
MS: Multi-step
NEAA: Non- essential amino acids
OPS: Open pulled straw
PBS: Phosphate Buffered Saline
PVA: Polyvinyl alcohol
SOF: Synthetic Oviductal Fluid
TS: Two-step
VS: Vitrification solution
