# Correction to: High *in vitro* survival rate of sheep *in vitro* produced blastocysts vitrified with a new method and device

**DOI:** 10.1186/s40104-019-0415-9

**Published:** 2019-12-17

**Authors:** Sergio Ledda, Jen M. Kelly, Stefano Nieddu, Daniela Bebbere, Federica Ariu, Luisa Bogliolo, Dity Natan, Amir Arav

**Affiliations:** 1 Department of Veterinary Medicine, University of Sassari, Sassari, Italy; 2 South Australian Research and Development Institute, Turretfield Research Centre, 129 Holland Road, Rosedale, SA 5350 Australia; 3 FertilSafe Ltd, 11 Haharash st, 7403118 Ness Ziona, Israel

**Correction to: J Anim Sci Biotechnol**

**https://doi.org/10.1186/s40104-019-0390-1**

In the original publication of this article [1], the author point out an error in Fig. 3. The correct Fig. 3 is below.

**Fig. 3:**
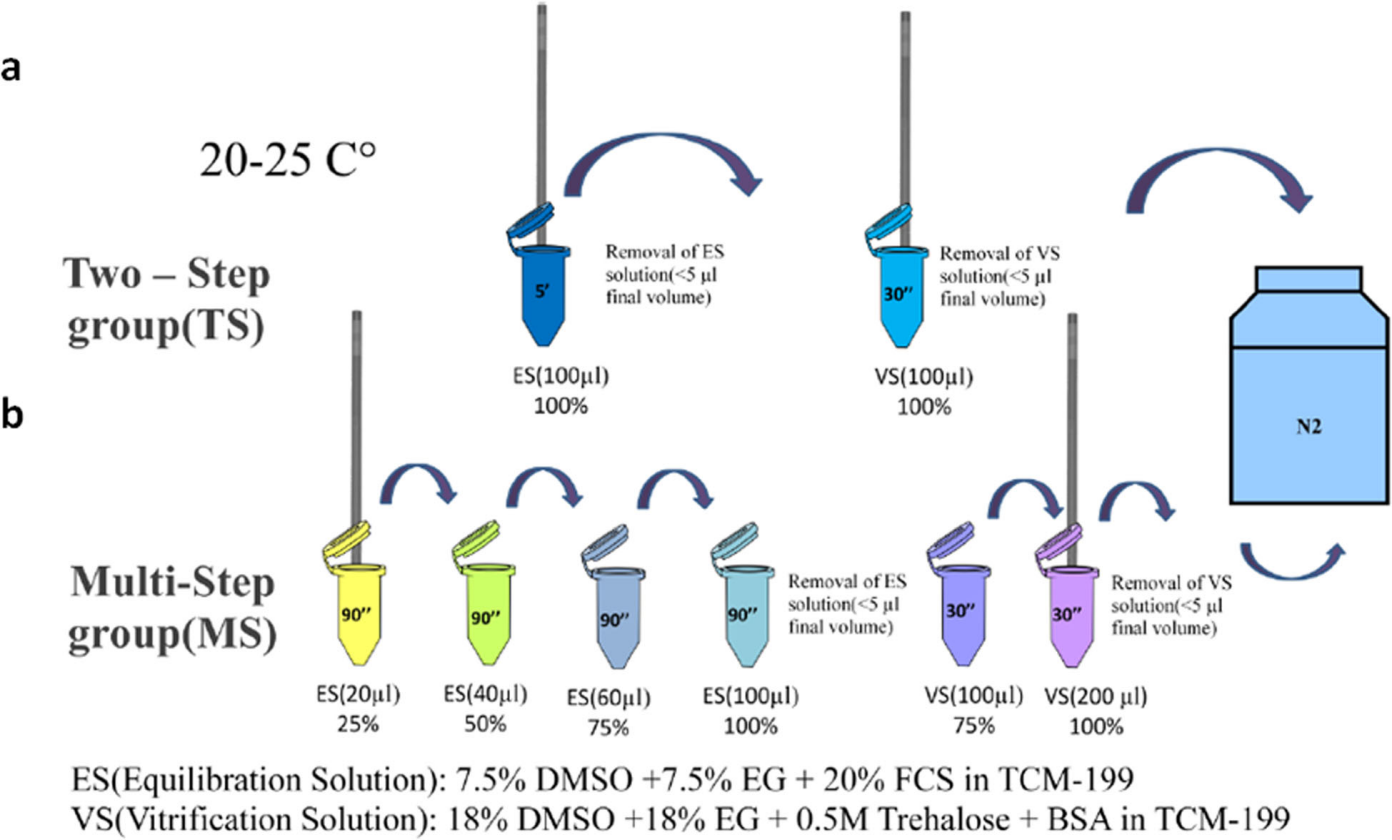
Flow chart of blastocyst vitrification using E.Vit device. **a** Two-step method **b** Multi-step method

The publisher apologizes to the readers and authors for the inconvenience.

The original publication has been corrected.
